# A review of recent advances in engineering bacteria for enhanced CO_2_ capture and utilization

**DOI:** 10.1007/s13762-022-04303-8

**Published:** 2022-06-20

**Authors:** H. Onyeaka, O. C. Ekwebelem

**Affiliations:** 1 School of Chemical Engineering, University of Birmingham, Edgbaston, Birmingham, B15 2TT UK; 2 Faculty of Biological Sciences, University of Nigeria, Nsukka, 410001 Nigeria

**Keywords:** Bacteria, CO_2_ capture, CO_2_ fixation, Microbial fixation, CO_2_ utilization, Microorganisms

## Abstract

Carbon dioxide (CO_2_) is emitted into the atmosphere due to some anthropogenic activities, such as the combustion of fossil fuels and industrial output. As a result, fears about catastrophic global warming and climate change have intensified. In the face of these challenges, conventional CO_2_ capture technologies are typically ineffective, dangerous, and contribute to secondary pollution in the environment. Biological systems for CO_2_ conversion, on the other hand, provide a potential path forward owing to its high application selectivity and adaptability. Moreover, many bacteria can use CO_2_ as their only source of carbon and turn it into value-added products. The purpose of this review is to discuss recent significant breakthroughs in engineering bacteria to utilize CO_2_ and other one-carbon compounds as substrate. In the same token, the paper also summarizes and presents aspects such as microbial CO_2_ fixation pathways, engineered bacteria involved in CO_2_ fixation, up-to-date genetic and metabolic engineering approaches for CO_2_ fixation, and promising research directions for the production of value-added products from CO_2_. This review's findings imply that using biological systems like modified bacteria to manage CO_2_ has the added benefit of generating useful industrial byproducts like biofuels, pharmaceutical compounds, and bioplastics. The major downside, from an economic standpoint, thus far has been related to methods of cultivation. However, thanks to genetic engineering approaches, this can be addressed by large production yields. As a result, this review aids in the knowledge of various biological systems that can be used to construct a long-term CO_2_ mitigation technology at an industrial scale, in this instance bacteria-based CO_2_capture/utilization technology.

## Introduction

At the end of the 21st Conference of the Parties to the United Nations Framework Convention on Climate Change in December 2015, 195 countries signed the Paris Agreement. The agreement intends to “strengthen the global response to the threat of climate change” by limiting global average temperature rises to “well below 2 degrees Celsius above pre-industrial levels.” (United Nations 2015). Carbon dioxide (CO_2_) emissions have been continuously increasing in recent years, and these worrying trends are expected to continue. The rise in the earth’s temperature started just at the dawn of the industrial age, resulting in an increase in the amount of the so-called Greenhouse Gases (GHG) such as CO_2_, CH_4_, N_2_O, and chlorofluorocarbons (Ekwebelem et al. 2020). Over 80% of the global energy production is made from the burning of fossil fuels (Barati et al. 2021; Olivier and Peters 2020). These industrial processes release a significant amount of CO_2_ (Omoregbe et al. 2020; Wang et al. 2020), which makes up 68% of the total emissions (Olivier and Peters 2020). However, these GHGs have played a fundamental role in maintaining our planet’s temperature and life as we know it today (Barati et al. 2021; Senatore et al. 2020). On the other hand, an increase in food demand production is another global challenge linked to carbon fixation (Gleizer et al. 2020). Furthermore, a global effort to minimize Carbon footprint necessitates the decarbonization of several global major industries (de Blas et al. 2020; du Pont et al. 2016). In this light, the extensive production of carbon–neutral fuels (e.g., biodiesel, bioethanol, biomethanol, hydrogen, etc.) for transportation and energy storage has been identified as a sustainable approach (de Blas et al. 2020; du Pont et al. 2016; Ekwebelem et al. 2020; Gleizer et al. 2020; Kumar et al. 2018; Obileke et al. 2021).

To mitigate this global challenge (CO_2_ emission), more sustainable strategies have been proposed such as advancing the energy efficiency of the current technologies to improve CO_2_ fixation and improving natural CO_2_ capturing effectiveness (Kumar et al. 2018). Interestingly, efforts in the form of large-scale Carbon Capture and Storage (CCS) projects (about 39) are ongoing worldwide following these proposed sustainable approaches (Budinis et al. 2018). Unfortunately, only 29 (the number had increased from 17 in 2018) are fully operational (Jaganmoha 2021), while the starting financial demands have also greatly limited its global development (Budinis et al. 2018). However, state-of-the-art developments in genetic engineering and membrane biotechnologies have today made it possible to tackle these economic barriers using the same microorganisms that have carried out carbon sequestration and fixation in the carbon cycle for decades (Pattharaprachayakul et al. 2020; Schweitzer et al. 2021; Zahed et al. 2021). This microbial CO_2_ sequestration and fixation aid by ribulose 1,5-bisphosphate carboxylase/oxygenase (RuBisCO) and Carbonic Anhydrase (CA) are common in both archaeal and bacterial domains (Hu et al. 2019; Saini et al. 2011; Salehizadeh et al. 2020). Through these advances, CO_2_ can be efficiently converted into biomass and useful compounds such as CO, CH_4_, CH_3_OH, DME, olefins, and higher hydrocarbons that can contribute significantly in protecting the ecosystem (De Vietro et al. 2019; Tursi 2019; Tursi et al. 2019).

Due to the current environmental challenges, utilizing CO_2_ as a bio-feedstock for sustainable food and fuel production is attracting immense interest. Moreover, the effectiveness of abiotic solutions for CO_2_ utilization is limited by low product selectivity (generating unwanted products), extreme condition requirements for full functionality, and specificity to the composition of the feedstock (Gleizer et al. 2020). Biotic solutions, on the other hand, can overcome these limitations because they require certain climate conditions and are very specialized and resilient to environmental changes and suspended particles in chemicals (Gleizer et al. 2020; Senatore et al. 2020). Therefore, another approach—synthetic biology—has been leveraged as a promising way of overcoming these challenges. Through this approach, microorganisms and biosynthetic pathways can be modified by linking two pathways that do not co-exist naturally or localizing a pathway to an organelle to enable improvement over some of their limiting natural components. Furthermore, synthetic biologists and molecular biologists have developed engineering tools capable of modeling and engineering organisms with an enhanced potential to fast-track the pace of innovation and bioprocess optimization (Antoniewicz 2019; Yadav 2021). Not surprisingly, our knowledge base in molecular and synthetic biology is expanding simultaneously with our knowledge on how to mitigate global CO_2_ emissions for a clean environment. Undeniably, it is imperative to continue the efforts of developing sustainable strategies for minimizing carbon footprint. This review discusses various techniques that have been utilized to enhance the ability of bacteria to capture and utilize CO_2_.

## Global CO_2_ emission trends

**Fig. 1:**
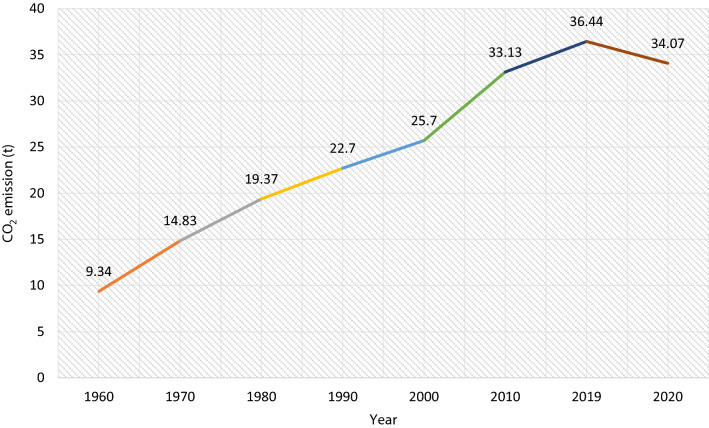
Global CO_2_ emissions trends in billion metric tons

Prior to around 459,000 years ago, the CO_2_ concentration in the atmosphere was consistently less than 260 parts per million volume (ppmv) (Data 2019; Lüthi et al. 2008; Pisaric and Smol 2021). However, between 660 and 670,000 years ago, this value reached its lowest value of 170ppmv. This value quickly grew during the industrial age, reaching 386ppmv in 2010, with an annualized rate of roughly 2ppmv (Data 2019). The highest known value is 419 ppmv, which was reported in June 2021 (NOAA 2021). The global CO_2_ emissions have been 1.6% higher in June 2021 than June 2020 (NOAA 2021). GHG emissions are currently 57% greater than in 1990 and 43 percent higher than in 2000 (excluding land-use change) (NOAA 2021). As shown in Fig. 1., there was a dramatic and progressive rise in CO_2_ emissions after the industrial revolution, from 9.34 billion metric tons in 1960 to 36.44 billion metric tons in 2019. Studies show that this continuous increase has been the case since the start of the second industrial age (Hashimoto 2019). The only recorded reduction in CO_2_ emission globally was in 2020, which is due to the global lockdown caused by the COVID-19 pandemic (Global Carbon Project 2020). The global lockdown led to a decrease in global emissions of greenhouse gases as well as those resulting from non-combustion.

CO_2_ emissions from fossil fuels are the most significant contributor to GHG, with China (10.06GT), USA (5.42GT), India (2.65GT), Russia (1.16GT), and Japan (1.16GT) being the major contributors (Global Carbon Project 2020). Other major contributors making up the top ten list are Germany (0.75GT), Iran (0.72GT), South Korea (0.65GT), Saudi Arabia (0.62GT), and Indonesia (0.61GT) (Global Carbon Project 2020). Since 1880, when global average temperature increases were first recorded, 2020 was by a narrow margin one of the six hottest years (2015–2020), effectively tying 2016, the previous record (NASA 2021). Just like in 2019, when temperatures were warmer than average globally, temperatures throughout Europe, the Middle East, parts of Asia, and New Zealand have reached new highs. (Global Carbon Project 2020; Yoro and Daramola 2020). Fortunately, carbon capture, also known as sequestration, is an effective strategy to scavenge carbon dioxide from the atmosphere (Hart and Onyeaka 2020). It has become an effective approach to mitigate global warming through carbon footprint reduction. In this way, GHGs released through natural and anthropogenic activities and accumulated in the ecosystem are slowed down. In light of this, Fig. 1 shows an updated worldwide CO_2_ emission trajectory for 2020, gathered from earlier studies (Fraccascia and Giannoccaro 2019; Greer et al. 2019a, 2019b; Holz et al. 2018; Zhang et al. 2019).

## Carbon capture and storage (CCS) and carbon capture and utilization (CCU) technologies

**Fig. 2:**
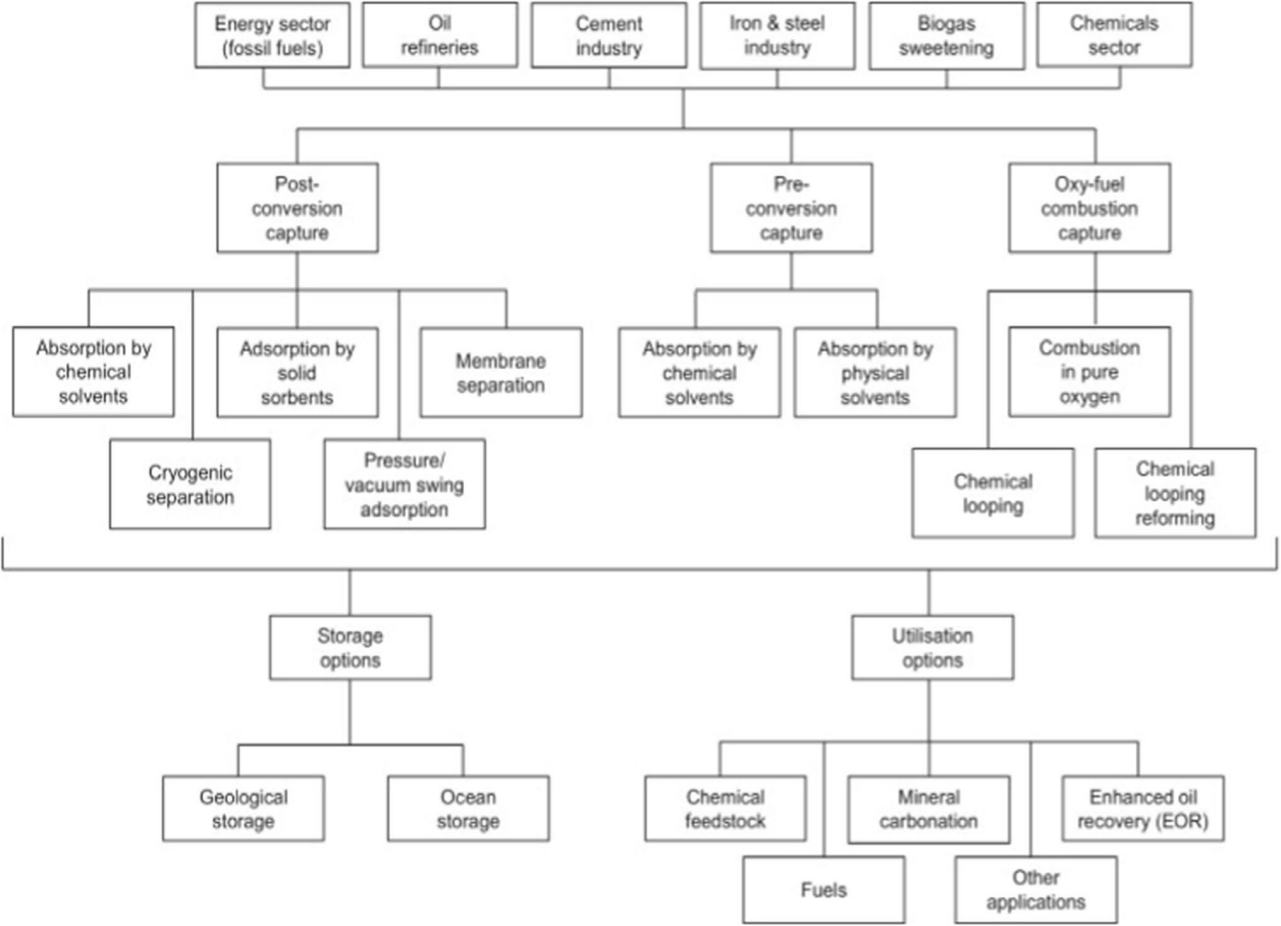
Different carbon capture, storage, and utilization options. Adapted from Cuellar-Franca & Azapagic

**Fig. 3:**
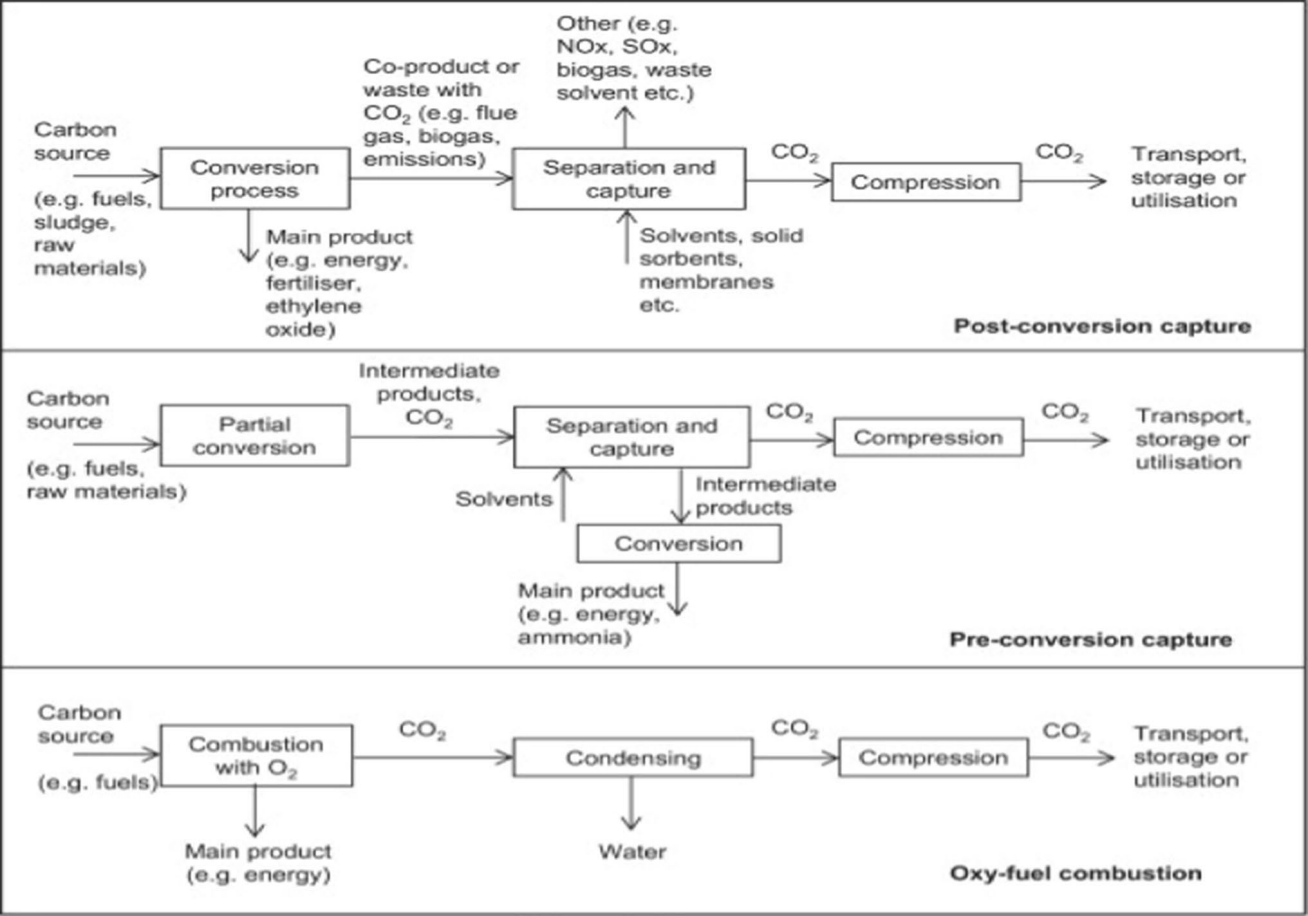
Carbon capture options. **Adapted from Singh et al. (**Singh et al. 2011**) and Zaimes and Khanna (**Zaimes and Khanna 2013**)**

Carbon capture and storage (CCS) and carbon capture and utilization (CCU) are technologies that capture emissions of CO_2_ from point sources (e.g., industrial operations and power plants) for storage to prevent them from being released into the atmosphere (Markewitz et al. 2012). The distinction between CCS and CCU lies in where the captured CO_2_ ends up. CCS involves transferring collected CO_2_ to a suitable location for prolonged storage (Markewitz et al. 2012; Weisser 2007; Zapp et al. 2012), while CCU involves converting captured CO_2_ into value-added products (Markewitz et al. 2012). Figure 2 summarizes the various CCS and CCU choices. Post-conversion, pre-conversion, and oxy-fuel combustion are the three CO_2_ capture alternatives (Singh et al. 2011; Zaimes and Khanna 2013). It should be noted that the purpose of this article is not to offer a comprehensive technical review of CCS and CCU technologies; rather, it is to provide context and perspective for the article's main goal, which is to review and analyze recent significant breakthroughs in engineering bacteria utilize CO_2_ and other one-carbon compounds as substrate. Figure 3 summarizes these points. Another possibility is CO_2_ fixation by biomass. Because of the need for biofuels, microalgae are now being employed for this purpose. As a result, this is potentially a CCU option rather than a CCS approach. This is because microalgae would not be cultivated only to capture CO_2_ (Cuéllar-Franca and Azapagic 2015). From an economic standpoint, CCU promises to be a better alternative than CCS because CCS is a non-profitable operation. However, to maintain a positive economic and environmental balance, CCU's cost-effectiveness and environmental implications must be closely evaluated (Cuéllar-Franca and Azapagic 2015). As previously stated, instead of storing, the captured CO_2_ can be applied in the production of as a commercial product, either directly or after conversion. CO_2_ may be used directly in the food and beverage sector, as well as for enhanced oil recovery (EOR); it can also be processed into chemicals or fuels (Cuéllar-Franca and Azapagic 2015). Other solutions include increased oil and coal-bed methane recovery, CO_2_ conversion to chemicals and fuels, mineral carbonation, microalgae-based biofuels, liquid fuel production, urea production and yield boosting, and many other avenues that involves the utilization of CO_2_ as a chemical feedstock (Bains et al. 2017). The main challenge to CCU becoming a climate mitigation potential is that there are limited possibilities to have a meaningful effect by offsetting merely 1% of yearly CO_2_ emissions in the United States (Aresta and Dibenedetto 2007). The energy-intensive process of CO_2_ conversion, regulated by thermodynamics, is the fundamental challenge. As a result, in certain situations, the link to renewable energy may be an all-too-quick contrast to its disadvantageous carbon balance (von der Assen et al. 2014). Nonetheless, CO_2_-based processing (von der Assen and Bardow 2014) is a less carbon-intensive current method. To resolve this issue, a thorough examination of the CO_2_-emitting industry is required to determine the entire scope of its implications.

(Cuéllar-Franca and Azapagic 2015).

## Bacteria growth for CO_2_ capture and utilization

It is well known that higher plants and microalgae possess the ability to fix CO_2_; however, bacteria have many benefits over these species, including a considerably quicker rapid growth rate and life cycle, the capacity to exist in a culture of high density, and the ability to be genetically engineered more readily (Bharti et al. 2014). Other than that, bacteria, just like other microbes, can produce a broad range of bio-alcohols and fatty acids for oil production which are essential industrial compounds (Bharti et al. 2014; Mohan et al. 2016).

The most popular industrial growing techniques for bacteria, like microalgae, are open ponds. Open ponds, comparatively, are a cost-effective culture system, however, it require a large surface area and are prone to contamination, which are two factors that are considered major limitations. Other more useful methods for bacteria growth for CCU are bioreactors and photobioreactors. Even for industrial applications, bioreactors and photobioreactors allow for the regulation of a broad variety of living conditions, but they are still highly costly equipment (Costa et al. 2006; Ketheesan and Nirmalakhandan 2012). Even though researchers have identified bacteria as a feasible option for CO_2_ capture from the atmosphere, the downside to their utilization involves the significant amount of work needed for their maintenance (Kumar et al. 2018; Saini et al. 2011). One of the most important qualities of this kind of system is advanced growth control settings, such as maintaining temperature and certain pH levels, controlling light, adding nutrients, and other environmental factors that prevent external contamination of pure cultures (Costa et al. 2006; Goli et al. 2016; Jajesniak et al. 2014). However, since bacterial cultures in open ponds systems are fragile, the most popular culture methods for these bacteria are flat panel photobioreactors and horizontal tube photobioreactors (Gebicki et al. 2009; Norsker et al. 2011). To effectively engineer a growth system for bacteria for CCU processes, a rectangular or square base frame is covered on both ends by a transparent panel in a flat panel photobioreactor. The level of aeration is maintained at 1L of air per liter of photobioreactor volume per minute (Sierra et al. 2008), and the photobioreactor is developed to take advantage of sunshine (Carvalho et al. 2006). Polyethylene, Polyvinyl Chloride (PVC or Vinyl), Poly(methyl methacrylate), or other acceptable materials with the correct thickness are used to ensure the gas tightness of the enclosed space, resist hydrostatic pressure, and decrease panel deflection (Gebicki et al. 2009; Norsker et al. 2011). A steel mesh frame binds the panels together to maintain the seal (Norsker et al. 2011). Flat panel photobioreactors are particularly attractive because when the thickness is maintained to a minimum, they have a high area-to-volume ratio which is favorable to bacteria growth (Carvalho et al. 2006). On the other hand, horizontal tubular photobioreactors are transparent tubular reactors with a certain inclination (less than 10 degrees). Interestingly, their orientation toward sunlight assists positively in a high light conversion efficiency (Dasgupta et al. 2010; Gebicki et al. 2009).

## CO_2_ capture and utilization by bacteria

Admittedly, the rise in CO_2_ amount, attributable to anthropogenic activities, has a serious effect on the ecosystem and there is an acknowledged need to develop technologies for the sustainable capture and utilization CO_2_ (Chu et al. 2017; Jiang et al. 2019; Mustafa et al. 2020; Vidales et al. 2019). As a result, the generation and usage of renewable energy have piqued people's curiosity (HASSOUN and Hicham 2020; Mikhno et al. 2021). Primarily, to achieve a carbon–neutral environment with a sustainable paradigm, the amount of CO_2_ emission should be equal to the amount used (Mohan et al. 2016; Senftle and Carter 2017). Fortunately, various methods, such as biological CO_2_ conversion using microbes (Chiranjeevi et al. 2019; Ghosh and Kiran 2017; Molitor et al. 2019; Sultana et al. 2016), chemo-catalytic CO_2_ conversion via organic or inorganic catalysts (Aresta et al. 2014; Taheri Najafabadi 2013), light-induced or electrocatalytic CO_2_ conversion (Hu et al. 2013; Ma et al. 2014; Tu et al. 2014), and catalytic hydrogenation of CO_2_ (Ashley et al. 2009; Chang et al. 2017; Rodemerck et al. 2013; Saeidi et al. 2014; Wang et al. 2018), have shown the capacity to convert CO_2_ to bio-based products. However, from a large-scale point of view, none of these novel methods can solely resolve CO_2_ capture and usage problems.

Just as autotrophic bacteria are innately wired to utilize CO_2_ as the sole carbon using light energy or inorganic compounds (Fry and Peel 2016), methylotrophic bacteria, on the contrary, have the potential to utilize reduced one-carbon compounds containing no carbon–carbon bonds (example formate, methanol, and other methylated compounds) as sole carbon sources and energy (Kumar et al. 2019b, 2016). Interestingly, these microbes can be modified genetically to enhance their suitability for bioproduction processes, even in industrial settings. A US company LanzaTech currently produces simple substrates like acetone, ethanol, and lactate from waste syngas and flue gas using acetogens, and autotrophic bacteria (Liew et al. 2016). In this gas fermentation process, CO_2_, CO, and H_2_ served as the carbon and reducing energy sources. Furthermore, several high-profile projects on CO_2_ capture at pilot or industrial size have been carried out and developed in many industrialized nations Italy (EniTecnologie), Germany (E’ON Hanse AG, Vattenfall’s Senftenberg), New Zealand (LanzaTech), Netherlands (Algaelink), United Kingdom (AlgaeCAT), Canada (Carbon2Algae Solutions Inc. and the Natural Research Council (NRC), China (Hearol project), USA (Touchstone Research Laboratory, GreenFuel Technologies, Agcore Technologies’ COPAS™) (Salehizadeh et al. 2020). At the laboratory scale, Sakimoto (Sakimoto et al. 2016) investigated the solar-to-chemical potential of a biological-inorganic hybrid (*Moorella thermoacetica* with cadmium sulfide nanoparticles) to generate acetic acid from CO_2_ under visible radiation. These appreciable findings suggested a self-replicating approach toward solar-to-chemical CO_2_ reduction via bacteria. Subsequently, various inorganic-biological hybrid systems for CO_2_ capture and utilization were developed. In the studies, the metabolic flexibility of the bacteria was adapted by incorporating light-harvesting inorganic materials to initiate the transformation of CO_2_ into bio-based commodities (Ding et al. 2019; Kumar et al. 2019a; Wang et al. 2019; Ye et al. 2019).

Recent work has shown that bacterial isolates (*Bacillus altitudinis*) from mangrove sediments in India with positive carbonic anhydrase (CA) activity, showed significant sequestering ability with a reduction of 97% CO_2_ (Nathan and Ammini 2019). In an earlier study, researchers genetically modified a lithoautotrophic Gram-negative bacteria (*Ralstonia eutropha*) to generate isobutanol and 3-methyl-1-butanol utilizing CO_2_ as the sole source of carbon and electricity as the only energy input (Li et al. 2012). Liu et al. coupled the same bacterium (*Ralstonia eutropha*) with a cobalt-phosphorus water-splitting catalyst in subsequent research to convert CO_2_ straight into biomass, biofuels, or other value-added products (Liu et al. 2016). Furthermore, owing to the simple growth requirement of Heterotrophs like *Escherichia coli,* some studies have explored its potential for efficient capture and conversion of CO_2_ and other one-carbon compounds. Interestingly, these studies shared a considerable similarity in their sources of energy, which are one-carbon compounds (formate or methanol) that can be produced via electrochemical reduction of CO_2_ (Marlin et al. 2018; Yishai et al. 2016). For example, Chen et al. explored the ability of engineered *E. coli* to grow on methanol which is a renewable one-carbon (C1) feedstock for microorganisms (Chen et al. 2020). By using the reductive glycine pathway, Kim et al. explored the growth of reprogrammed *E. coli* on formate and methanol as a sustainable bioproduction rooted in CO_2_ and renewable energy (Kim et al. 2020). Also, Gleizer et al. studied the potential of *E. coli* to generate all biomass carbon from CO_2_ conversion (Gleizer et al. 2019).

Notwithstanding these revolutionary advances, some downsides such as poor multiplication rates, inadequate characterization, and incomplete validation at the industrial level have currently made these strains unsuitable for utilization at an industrial scale (Gleizer et al. 2020). Interestingly, Gleizer et al. (Gleizer et al. 2020) highlighted that the capacity of these modified microbes to generate energy from one-carbon compound, when combined with the electrochemical conversion of CO_2_ to one-carbon compound, increases the opportunities for a carbon–neutral economy. Another innovative approach is the use of hybrid systems. Hybrid systems are biotic-abiotic technologies that combine the best of both worlds and it is predicted that they will eventually outperform photosynthesis in terms of yields and energy effectiveness (Gleizer et al. 2020). For instance, a newly created hybrid microbe–metal interface integrates an inorganic, semiconducting light-harvester material with efficient and simple bacteria to create a revolutionary metal–microbe interface that aids microorganisms indirectly in capturing energy from the sun (Sahoo et al. 2020). Further, Su and colleagues studied the efficiency of nanowire-bacteria hybrids for Solar-powered CO_2_ Fixation (Su et al. 2020). They were able to enhance the CO_2_-reducing efficiency in a silicon nanowire/*Sporomusa ovata* system by looking into the microorganism-cathode interface. The rate of CO_2_ reduction at high voltage was inherently limited by a poor bacterium nanowire interface caused by an unfavorable alkaline environment (Su et al. 2020). In this study, the creation of a close-packed nanowire-bacteria cathode was aided by adjusting the bulk electrolyte pH and improving its buffering volume (Su et al. 2020).

It has been recommended that photovoltaic cells possess a greater energy conversion efficiency than photosynthesis in producing H_2_ and CO as feedstocks for archaea (Gleizer et al. 2020). These technologies are fundamentally modular, offering the selection of a biological host and a source of energy independent of one another (Gleizer et al. 2020). Similarly, the use of genetically tractable microorganisms is another appealing technology because it makes it easier to introduce new pathways (Gleizer et al. 2020). For instance, using H_2_ as a source of energy in *E. coli,* H_2_ is generated more effectively than formate and is suitable for the growth of microorganisms (Claassens et al. 2018). In the same token, genetically tractable hosts can also be used to introduce novel biosynthetic pathways to generate value-added products (Pontrelli et al. 2018). While these scientific achievements have been referred to as a “milestone,” there is still a long way to go, as it will be a few years before we can see this microorganism in action at industrial scale (Callaway 2019).

## Engineering approach to improving CO_2_ capture by bacteria

Recent technological advances in comprehending microbial metabolic pathways, decoding genetic makeups, and much more have revolutionized the way we unravel the code of life, allowing us to make modifications that were seemingly unimaginable (Jiang et al. 2021; Lee et al. 2012; Majidian et al. 2018; Park et al. 2018b). Chemicals can be divided into four categories if they are found or reported to exist in nature “natural vs. non-natural,” and whether or not they can be manufactured by microbes' pathways “inherent vs. noninherent”: i) natural-inherent chemicals; (ii) natural-noninherent chemicals; (iii) nonnatural-noninherent chemicals; and (iv) nonnatural-created chemicals (Lee et al. 2012). Metabolic engineers analyze not just the effectiveness of a proposed metabolic pathway but also the most efficient means of constructing it in their efforts to obtain these many categories of molecules. Natural-inherent compounds, for example, can frequently be overproduced by directly altering the host strain to maximize native pathway fluxes at the system level (Lee et al. 2012). Consequently, microbial metabolic pathway engineering may concentrate on more intuitive approaches that employ standard metabolic and bioprocess engineering approaches to solve well-defined and well-known challenges (Lee et al. 2012).

Bacteria, namely *E. coli*, are one of the first genetically modified prokaryotic organisms (Li et al. 2015; Yang et al. 2020). This microorganism demonstrates a wide range of mutations as a result of the application of physical or chemical mutagens that will be chosen (Choi et al. 2016; Jajesniak et al. 2014; Li et al. 2015; Yang et al. 2020). This is because of their fast growth feature and the selective media on which they are cultured (Jajesniak et al. 2014). For example, after overnight growth, *E. coli* generates approximately 10^9^ cells per milliliter (U/mL) (Senatore et al. 2020). Up to this point, only six CO_2_ fixation pathways have been suggested: (i) the Calvin–Benson–Bassham (CBB) cycle; (ii) the 3-hydroxypropionate/4-hydroxybutyrate cycle (3HP-4-HB); (iii) the dicarboxylate/4-hydroxybutyrate (DC/4-HB) cycle; (iv) the 3-Hydroxyproppionate bicycle (3-HP/malyl-CoA cycle); (v) the reductive tricarboxylic acid (rTCA) cycle and; (vi) the Wood–Ljungdahl (WL) (Saini et al. 2011; Salehizadeh et al. 2020). The aerobic pathways include the CBB, 3HP-4HB, and 3-HP/malyl-CoA, whereas the anaerobic pathways are the rTCA, WL, and DC/4HB (Saini et al. 2011; Salehizadeh et al. 2020).

A lot of work has recently gone into creating potential CO_2_ fixation pathways utilizing synthetic biology (Gong et al. 2016), and protein and metabolic engineering (Zhou et al. 2016). Synthetic biology concentrates on redesigning and repositioning innate pathways for CO_2_ fixation, modifying CO_2_-fixation pathways to increase CO_2_ delivery, and developing and optimizing the efficiency and durability of CO_2_ fixation enzymes to enable effective CO_2_ fixation (Gong et al. 2016, 2018). In a proof-of-concept experiment seeking to overhaul *E. coli*’s diet, Antonovsky and colleagues (Antonovsky et al. 2016) successfully introduced the ability to synthesize biomass from CO_2_ into *E. coli*, a heterotrophic organism*.* They developed a strain that absorbed CO_2_; however, it only represented a minute fraction of the organism's carbon intake; the remainder originated from an organic substance called pyruvate, which was supplied via a non-native Calvin–Benson–Bassham (CBB) cycle in evolved *E. coli* (Antonovsky et al. 2016).

In another latest work, metabolic rewiring and directed evolution generated *E. coli* strains that utilize CO_2_ as its primary source of carbon, with formate being oxidized to meet all of the reducing power and energy requirements via non-native CBB cycle (Gleizer et al. 2019). This led to the successful rewiring of obligate heterotrophs to full autotrophy over laboratory timescales (Gleizer et al. 2019). When compared to regular *E. coli*, which may grow exponentially every 20 min, autotrophic *E. coli* are slackers, multiplying every 18 h when cultivated in a 10% CO_2_ atmosphere (Gleizer et al. 2019). Hence, the emerging picture suggests that they cannot live without sugar at the current CO_2_ levels in the atmosphere, which are 0.041 percent. In trying to understand the genetic basis underlying this metabolic transition, Herz et al. suggest that five mutations are enough to permit robust growth when a non-native CBB cycle supplies all the metabolic building blocks derived sugar (Herz et al. 2017). These mutations can be discovered in enzymes (*prs*, *serA*, and *pgi*) that impact the efflux of intermediates from the autocatalytic CO_2_ fixation cycle to biomass or in critical regulators (*crp* and *ppsR*) of carbon metabolism (Herz et al. 2017).

More studies have also demonstrated an admirable example of carbon metabolism plasticity in carbon-fixing bacteria. In *Synechococcus elongatus* PCC 7942, the CBB cycle and Embden–Meyerhof pathway were engineered to enhance carbon flow in favor of CO_2_ fixation. The hexose monophosphate shunt (HMP Shunt) pathway was reconfigured to increase ribulose-5-phosphate (Ru5P) as a precursor to CO_2_ fixation, which improved glucose metabolism (Kanno et al. 2017). To increase ribulose-5-phosphate to ribose-1,5-bisphosphate conversion and regulate cyanobacteria’s carbon metabolism, part of the operator gene (*cp12*) in the CBB cycle was removed. In the absence of light, this resulted in increased synthesis of 2,3-butanediol (Kanno et al. 2017). In another case, a biosystem coupling *Acetobacterium woodii* (an acetogen) with *Acinetobacter baylyi* ADP1 (a non-native alkane producer) designed for alkane production was proven by Lehtinen et al. (Lehtinen et al. 2018). In their study, nine synthetic two-step alkane biosynthesis pathways were designed and produced in *A. baylyi* using a combination of aldehyde- and alkane-producing enzymes. Although the generation of drop-in liquid fuels from CO_2_ was shown, the modular system's alkane productivity remained low, posing a huge research challenge in the future (Lehtinen et al. 2018). Moving forward, these innovations would result to lower emissions than those produced using traditional techniques, and they could even remove the CO_2_ from the atmosphere. Moreover, recent advances are now concentrating on the biotechnological enhancement of cyanobacteria and microalgae cultivation through biofilm Photobioreactors (PBRs) as a sustainable alternative to cut the cost of production at the industrial scale. PBRs have the benefits of requiring less water and having a comparatively simple harvesting process (Cheng et al. 2019; Guo et al. 2019). Table 1 summarizes some of the recent advancements in CO_2_-fixing engineered bacteria.

**Table 1:** Examples of bacteria that have been engineered to increase their CO_2_-fixation ability

Bacterial strain	Features	References
*Moorella thermoacetica*	Demonstrates a self-replicating pathway toward light-to-chemical CO_2_ reduction by selectively producing acetic acid from CO_2_	(Sakimoto et al. 2016)
*Ralstonia eutropha* H16	Expresses electricity-driven bioconversion of CO_2_ to isobutanol and 3-methyl-1-butanol	(Li et al. 2012)
*Ralstonia eutropha*	In the presence of O_2_, the rewired strain synthesizes biomass, fuels, or chemical compounds from lower CO_2_ concentrations	(Liu et al. 2016)
*E. coli* BW25113	Genetically reprogrammed *E. coli* grow effectively with methanol as the only source of carbon	(Chen et al. 2020)
*E. coli*	Rewired strain capable of growth on formate, methanol, and CO_2_	(Kim et al. 2020)
*E. coli*	The new strain coexpressed rubisco and phosphoribulokinase with formate dehydrogenase to allow CO_2_ fixation and reduction	(Gleizer et al. 2019)
*Sporomusa ovata*	Silicon nanowire/*Sporomusa ovata* system showed a high CO_2_-reducing rate and solar-driven CO_2_ fixation with high solar-to-acetate conversion	(Su et al. 2020)
*E. coli*	Evolved strain synthesized sugars from CO_2_ via non-native CBB pathway	(Antonovsky et al. 2016)
*Rhodobacter sphaeroides* MBTLJ-8	The rewired strain originates from *R. sphaeroides* 2.4.1. and has a higher CO_2_ fixing rate	(Park et al. 2018a)
*E. coli* BL21	Expresses the carbonic anhydrase gene originating from *Synechococcus sp.* PCC7002	(Gong et al. 2015)
*Moorella thermoacetica*	The *M. thermoacetica*/AuNC hybrid system harvests sunlight effectively allowing for continuous CO_2_ fixation	(Zhang et al. 2018)
*E. coli* BA207	Pyruvate carboxylase and nicotinic acid phosphoribosyltransferase are coexpressed in the new strain	(Liu et al. 2013)
*Rhodopseudomonas palustris*	The remodeled strain exhibited light-driven CO_2_ reduction to methane	(Fixen et al. 2016)

## Future prospects

A few of the problems facing researchers trying to address the problem of excessive CO_2_ emissions associated with a rise in greenhouse gases is the capacity to enhance CO_2_ conversion using the same natural mechanisms that have been used for ages to fix inorganic carbon sources. The sole economic drawback is currently related to cultivation techniques, which might be mitigated by excellent production rates due to genetic engineering methods (Senatore et al. 2020). Fortunately, metabolic engineering steps to enhance CO_2_ fixation via rewiring of central metabolisms, like i) splitting RuBisCO 's catalysis among many enzymes; ii) substituting the CBB cycle with other pathways; and; iii) replacing Rubisco with alternative carboxylation reaction, have the potential to transform CO_2_ fixation in the long run (Antonovsky et al. 2016; Bar-Even 2018; Claassens 2017; Herz et al. 2017; Hing et al. 2019). Albeit the CBB cycle is the most well-known pathway for photosynthetic CO_2_ fixation cycle by microorganism (Andorfer and Drennan 2021; Antonovsky et al. 2017), a potential synthetic pathway known as Malonyl-coA-Oxaloacetate-Glyoxylate (**MOG**) was reported as a better alternate to autotrophs' inherently poor CO_2_ fixation pathways (Salehizadeh et al. 2020). In comparison to the CBB cycle, the MOG pathway uses quicker carboxylases (e.g., phosphoenolpyruvate carboxylase or pyruvate carboxylase). These enzymes are highly oxygen-tolerant and have reduced ATP costs (Salehizadeh et al. 2020). Furthermore, the rapid advances in metabolic engineering techniques involving genome-scale modeling and sequencing of bacteria and yeast (*E. coli* and *Saccharomyces cerevisiae*), might present a whole new field in CO_2_ fixation by heterotrophic bacteria. Interestingly, with the aid of Maximum Driving Force (MDF), it is very possible now to identify thermodynamically viable metabolic pathways and even assess the CO_2_ fixation capability of heterotrophs such as *E. coli* via the innovative *OptMDFpathway*, particularly in the case of cell factories' metabolic design (Hädicke et al. 2018; Kanno et al. 2017; Savakis et al. 2015; Tabita 2005; Tracy et al. 2012). Now it is left for biotechnological engineering to tackle the exciting issue of increasing the efficiency of producing fuel, commodities, and food from CO_2_. Not surprisingly, the absence of infrastructure for manufacturing and storing hydrogen from water, as well as safety concerns and capital intensity, limits the utilization of CO_2_ into fuels. New manufacturing strategies including the usage of a succession of biological stages employed in contemporary biorefinery projects are still needed. This might be an intriguing method for implementing and developing new CO_2_ capture and conversion systems by bacteria.

## Conclusion

CO_2_ capture by bacteria is an appealing option for climate change mitigation and immediately creating bio-based commodities with added value from CO_2_. However, to extend the production of these valuable commodities from CO_2_, revolutionary innovations encompassing major biotechnological methods (synthetic biology, and metabolic and genetic engineering) will need to be used and further developed. In this review, various novel genetic engineering and synthetic approaches employed in the engineering of bacteria for improved CO_2_ capture and utilization were discussed. With a growing emphasis on climate remediation, the use of bacteria targeted at a severe lowering in the addition of value to the carbon dioxide extracted, the use of waste raw materials, and footprint will be the ones to watch in the future. The emerging picture from this review highlights the need for future studies should focus on the selection of efficient bacteria, genetically engineering alterations as well as designing and building synthetic metabolic pathways. Doing so will help to reduce the cost of production of value-added bio-based products via CO_2_ capture and conversion by bacteria. Finally, integrating bacteria CO_2_ fixation in addition to other industrial operations such as treatment of exhaust gas and wastewater, refining biogas, and direct manufacture of commodities from CO_2_ might be more productive. This might help address the primary ecological issues of global warming while also reducing the usual cost and performance constraints in microbiological CO_2_ capture and conversion on a large scale as well as technological advances.

## Data Availability

Not applicable.
